# Beta-defensin derived cationic antimicrobial peptides with potent killing activity against gram negative and gram positive bacteria

**DOI:** 10.1186/s12866-018-1190-z

**Published:** 2018-06-05

**Authors:** Ming Yang, Chunye Zhang, Michael Z. Zhang, Shuping Zhang

**Affiliations:** 1 Department of Veterinary Pathobiology, College of Veterinary Medicine, University of Missouri, Columbia, MO 65211 USA; 2 Department of Biomedical Science, College of Veterinary Medicine, University of Missouri, Columbia, MO 65211 USA; 3 Veterinary Medical Diagnostic Laboratory, College of Veterinary Medicine, University of Missouri, Columbia, MO 65211 USA

**Keywords:** Cationic antimicrobial peptides, Peptide design, Salt resistance, Antimicrobial activity, Multidrug-resistant *Pseudomonas aeruginosa*, Methicillin-resistant *Staphylococcus pseudintermedius*

## Abstract

**Background:**

Avian β-defensins (AvBD) are cationic antimicrobial peptides (CAMP) with broad-spectrum antimicrobial activity, chemotactic property, and low host cytotoxicity. However, their bactericidal activity is greatly compromised under physiological salt concentrations which limits the use of these peptides as therapeutic agents. The length and the complex structure involving three conserved disulfide bridges are additional drawbacks associated with high production cost. In the present study, short linear CAMPs (11 to 25 a.a. residues) were developed based on the key functional components of AvBDs with additional modifications. Their biological functions were characterized.

**Results:**

CAMP-t1 contained the CCR2 binding domain (N-terminal loop and adjacent α-helix) of AvBD-12 whereas CAMP-t2 comprised the key a.a. residues responsible for the concentrated positive surface charge and hydrophobicity of AvBD-6. Both CAMP-t1 and CAMP-t2 demonstrated strong antimicrobial activity against *Pseudomonas aeruginosa*, *Staphylococcus aureus* and *Staphylococcus pseudintermedius*. However, CAMP-t1 failed to show chemotactic activity and CAMP-t2, although superior in killing *Staphylococcus* spp., remained sensitive to salts. Using an integrated design approach, CAMP-t2 was further modified to yield CAMP-A and CAMP-B which possessed the following characteristics: α-helical structure with positively and negatively charged residues aligned on the opposite side of the helix, lack of protease cutting sites, C-terminal poly-Trp tail, N-terminal acetylation, and C-terminal amidation. Both CAMP-A and CAMP-B demonstrated strong antimicrobial activity against multidrug-resistant *P. aeruginosa* and methicillin-resistant *S. pseudintermedius* (MRSP) strains. These peptides were resistant to major proteases and fully active at physiological concentrations of NaCl and CaCl_2_. The peptides were minimally cytotoxic to avian and murine cells and their therapeutic index was moderate (≥ 4.5).

**Conclusions:**

An integrated design approach can be used to develop short and potent antimicrobial peptides, such as CAMP-A and CAMP-B. The advantageous characteristics, including structural simplicity, resistance to salts and proteases, potent antimicrobial activity, rapid membrane attacking mode, and moderate therapeutic index, suggest that CAMP-A and CAMP-B are excellent candidates for development as therapeutic agents against multidrug-resistant *P. aeruginosa* and methicillin-resistant staphylococci.

## Background

The rapid emergence and spread of antimicrobial resistance, particularly those associated with *Pseudomonas aeruginosa* and *Staphylococcus* spp*.*, have become a serious threat to public health [1, 2]. The Centers for Disease Control and Prevention (CDC) estimated that each year in the United States there are approximately 88,000 cases and 11,000 deaths due to infections with methicillin-resistant *Staphylococcus aureus* (MRSA) [3]. Various studies have been conducted to search for new classes of antimicrobial therapeutic agents or antibiotic alternatives with novel targets and modes of action [4]. Host cationic antimicrobial peptides (CAMPs), including linear peptides, α-helical peptides, circular and complex structures with loops and β-sheets constitute the first line of innate defense against microbial pathogens [5]. The features shared by these CAMPs are net positive charge and amphipathicity [6]. The cationic property of CAMP allows for the initial interaction of the peptide with the anionic surface groups of the microbial membrane and the hydrophobicity enables the peptide to integrate into the hydrophobic core of the membrane. The mechanism of action of CAMPs is complex, achieved primarily through membrane damage and possibly subsequent interactions with cellular machineries, and the potential for development of microbial resistance is low [6]. A major group of CAMPs with broad-spectrum antimicrobial activity is β-defensins which contain three cysteine-cysteine disulfide bridges [7]. In addition to their antimicrobial activity and low potential for the development of resistance by bacteria, β-defensins have several other beneficial characteristics, such as modulating host immune response (e.g. chemo-attracting immune cells) [8–11]. Our previous studies show that avian β-defensins (AvBDs) such as AvBD-6 and AvBD-12 possess the following biological properties: broad-spectrum antimicrobial activity, LPS-neutralizing ability, chemotactic activity, and minimal cell cytotoxicity [12–14]. Although β-defensins represent potentially a novel class of antimicrobial therapeutic agents, several obstacles must be overcome, including host cell cytotoxicity, degradation by proteases, loss of antimicrobial activity in the presence of a physiological concentration of salts, and high production cost due to their complex structure [15].

Via the characterization of the structure-function relationship of AvBDs and various analogues, it has been identified that the concentrated surface net positive charge and the N-terminal α-helix and the β2-β3 loop structure are essential functional domains for antimicrobial and chemotactic properties [13]. Linear AvBD analogues with a high net positive charge (+ 9) and an N-terminal helix-loop possess improved antimicrobial potency and partial chemotactic activity, compared to the wild-type AvBD-12 [13]. However, the linear AvBDs designed in our previous study are still sensitive to physiological salt conditions and the length of the peptides (45 amino acid residues) remain to be shortened to control the manufacturing cost. In addition, a previous study has indicated that linear peptides are more susceptible to protease degradation due to lack of complex tertiary structure stabilized by disulfide bridges found in natural defensin peptides [16].

To increase salt- and protease-resistance, several solutions have been proposed, including: incorporating nonproteinogenic amino acids (e.g. D-amino acid substitutions and bulky amino acid β-naphthylalanin) [17, 18] or LPS binding peptide motif (β-boomerang motif GWKRKRFG) [19], modifying the terminal regions via covalent linkage of a hydrophobic moiety (e. g. a sterol or a fatty acid) [20, 21], peptidomimetic [22], altering the structure, charge, hydrophobicity, and shortening the length of the peptide [23, 24]. These strategies successfully improved the antimicrobial function of CAMPs, but resulted in elevated hemolytic activity and increased manufacturing cost [18, 25]. In the present study, an integrated approach was utilized to design short and compositionally simple CAMPs with potent antimicrobial activity, improved resistance to salts and proteases and minimal cytotoxicity to host cells. The antibacterial property of the newly designed CAMPs against *P. aeruginosa* and *Staphylococcus* spp., including clinical isolates of multidrug-resistant *P. aeruginosa and methicillin-resistant S. pseudintermedius* (MRSP) was assessed under various conditions.

## Methods

### Bacterial strains and cultures

*Pseudomonas aeruginosa* (*P. aeruginosa*, ATCC 27853) and *Staphylococcus aureus* (*S. aureus*, ATCC 29213) were used to evaluate the novel CAMPs’ antimicrobial activity, salt- and protease-resistance, and membrane permeability. Ten multiple-drug resistant *P. aeruginosa* and ten methicillin-resistant *S. pseudintermedius* (MRSP) clinical isolates (Table 2) were used to evaluate the antimicrobial efficacy of CAMPs. The clinical isolates were cultured from diagnostic specimens by the microbiology staff at the University of Missouri Veterinary Medical Diagnostic Laboratory as part of standard service. The isolates were donated to the present project with appropriate permission. All bacterial strains were maintained and grown in Luria-Bertani broth or agar (LB, BD Difco™) at 37 °C as described previously [13, 14].

### Peptide synthesis and characteristics

All peptides were custom synthesized using the standard solid phase 9-fluorenylmethoxycarbonyl (Fmoc) method as previously synthesizing wild-type AvBDs [14] and purified by reverse phase high-performance liquid chromatography (RP-HPLC) (Lifetein, Hillsborough, NJ). The purity of the synthetic CAMPs was greater than 98.5% as verified by liquid chromatography-mass spectrometry (LC-MS) (Lifetein, Hillsborough, NJ). The charge and hydrophobicity of the newly designed CAMPs at neutral pH were calculated using online Peptide property calculator (PepCalc.com). Protease cutting sites were predicted by using PROSPER (https://prosper.erc.monash.edu.au) and SignalP 4.1 server (http://www.cbs.dtu.dk/ services/SignalP/). The three-dimensional structures of AvBDs and the newly designed templates were analyzed by using the I-TASSER (Iterative Threading Assembly Refinement) protein structure and function prediction program (http://zhanglab.ccmb.med.umich.edu/I-TASSER). The distribution of selected amino acid residues was evaluated using PyMOL, a user-sponsored molecular visualization system (https://www.pymol.org/). The helical wheel projection was calculated using the Helical Wheel Projections program (http://rzlab.ucr.edu/scripts/wheel/wheel.cgi).

### Circular dichroism spectrum analysis

Peptide structures were examined by far-UV circular dichroism (CD) spectroscopy with an Aviv Model 62DS spectrometer (Lakewood, NJ), in the wavelengths ranging from 190 to 250 nm using a path length of 1 mm. The spectra of peptides were measured at a concentration of 0.15 mg/ml in water. Spectra were baseline corrected by subtracting a blank spectrum containing only buffer and expressed as molar ellipticity θ (deg·cm^2^·mol^− 1^).

### Membrane permeabilizing assay

The membrane permeabilizing ability was determined using the propidium iodide (PI) uptake assay [26]. PI is a fluorescent molecule that can only penetrate the impaired microbial membrane and intercalate double-stranded DNA. PI staining was done according to the manufacturer’s instruction (Sigma Aldrich). In brief, the mid-logarithmic culture of *P. aeruginosa* (ATCC 27853) was harvested by centrifugation at 1000×*g* for 10 min and resuspended in PBS (1 × 10^8^ CFU/ml). The bacteria were treated with each CAMP at a concentration of 1 × MIC for 15, 30, 60, and 90 min, respectively. After the addition of PI, the suspension was further incubated for 5 min at room temperature and shielded from light. The bacterial mixture was coated on a microscope slide for analysis of red fluorescence. Images were captured using a Nikon fluorescent microscope with Olympus DP2-BSE software (ECLIPSE E600, Japan) and the number of fluorescent cells per field was counted by the ImageJ software (NIH, Bethesda, MD). The assay was performed in triplicate.

### Antimicrobial activity assay

Minimum inhibitory concentrations (MICs) were determined primarily based on the guidelines of the Clinical and Laboratory Standards Institute (CLSI) [27, 28]. The Muller Hinton (MH) II broth used in MIC assay contained 20–25 mg/L of calcium and 10–12.5 mg/L of magnesium. The procedures were described in previous studies [13, 14]. In brief, two-fold serially diluted CAMPs (2 to 256 μg/ml) were mixed with appropriate bacterial strains at a final concentration of 5 × 10^5^ CFU/ml in a 96-well microtiter plate (Nunc™, Thermo Fisher Scientific). Following incubation at 37 °C for 24 h, MIC was recorded. All assays were conducted in triplicate.

### Salt resistance assay

A major hindrance to clinical application of defensin peptides is the interference of function by cationic salts, often referred to as salt sensitivity [29]. The effect of salt on antimicrobial activity of novel CAMPs against *P. aeruginosa* ATCC 27853 and *S. aureus* ATCC 29213 was determined by a colony count assay as described previously [14], in the presence of either 0, 50, 100, and 150 mM NaCl or 0, 0.5, 1, and 2 mM CaCl_2_. Two peptide concentrations, 0.5 × MIC and 1 × MIC, were included. Medium without CAMP served as a negative control. Percent of killing was calculated using the following formula: (CFU_control_ - CFU_treated_) / CFU_control_ × 100% [14]. All assays were performed in triplicate.

### Hemolytic assay

The hemolytic assay was performed as described previously [25]. Briefly, mouse red blood cells (RBCs, Innovative Research, Novi, MI) were washed three times with phosphate-buffered saline (PBS, pH 7.4), centrifuged at 1000×*g* for 10 min, and resuspended in PBS to 10% (*v*/v). The RBCs were treated with CAMPs at various concentrations ranging from 4 to 512 μg/ml (2-fold serial dilutions) at 37 °C for 1 h. PBS and 0.2% Triton X-100 were used as negative and positive controls, respectively. The supernatant was transferred to a 96-well flat-bottomed polystyrene plate (Thermo Fisher Scientific), and the amount of hemoglobin released into the supernatant was determined by measuring the absorbance with a spectrophotometer at 540 nm. Hemolytic activity was expressed as the percentage of hemolysis and calculated using the following equation: hemolysis (%) = (A_s_-A_0_)/(A_100_-A_0_) × 100, where A_s_ is the absorbance of the sample, A_100_ is the absorbance of completely lysed RBCs in 0.2% Triton X-100, and A_0_ is the absorbance in the complete absence of hemolysis (PBS treatment). The assay was performed in triplicate. The therapeutic index (T.I.) was calculated according to a previously published formula: T.I. = MHC / MIC_GM_ [30]. MHC was the minimum hemolytic concentration that caused 5% hemolysis of mouse RBCs. The MIC_GM_ was the minimum inhibitory concentration of the peptide concentrations against bacterial growth after the geometric mean was calculated. MIC_G-_ was the MIC_GM_ for Gram-negative bacteria; MIC_G+_ was the MIC_GM_ for Gram-positive bacteria.

### Cell cytotoxicity assay

The cytotoxicity of CAMPs to JASWII (ATCC CRL-11904) and CHO-K1 (ATCC CCL-61) cells was determined using MTT (3-(4, 5-dimethylthiazol-2-yl)-2, 5-diphenyltetrazolium bromide, Thermo Fisher Scientific) a cell proliferation assay as described previously [14]. The following peptide concentrations were included in the present study: 64, 128, 256, and 512 μg/ml. Following CAMP treatment, percent of viable cells, relative to the untreated control, was recorded. The assays were performed in triplicate.

### Chemotaxis assay

CAMP-induced migration of JAWSII (ATCC CRL-11904) and CHO-K1 (ATCC CCL-61) transfected with CCR-2 was determined using a microchemotaxis assay described previously [14, 31]. Chemotactic indexes (C.I.) was calculated as the number of migrated cells induced by CAMPs divided by the number of migrated cells in the control wells without CAMPs. The assays were repeated five times.

### Protease resistance assay

Protease resistance was evaluated by using sodium dodecyl sulfate-polyacrylamide gel electrophoresis (SDS-PAGE) followed by antimicrobial assays. CAMP peptides (10 μg) were treated with various proteases at various concentrations comparable to or higher than that found in host or bacterial culture, including 0.12, 0.6, and 1.2 μg/ml of α-chymotrypsin (Thermo Fisher Scientific) [25], 0.4, 4.4, and 20 μg/ml of matrilysin (metalloproteinase-7, Sigma Aldrich) [32], 0.2, 2, and 20 μg/ml elastase (Thermo Fisher Scientific) [33], or 0.2, 2, and 20 μg/ml of cathepsin B (Thermo Fisher Scientific) [34]. Protease digestion assay was carried out in 20 μl of digestion buffer (25 mM Tris and 150 mM NaCl, pH 7.8) for 1 h at 37 °C. After treatment, the digestion mixture was analyzed by SDS-PAGE on 16.5% polyacrylamide gel. To determine the effect of protease digestion on the antimicrobial activity of two most active CAMPs, each peptide was first treated with a protease for 1 h at 37 °C. The mixture was then diluted to 1 × MIC of the peptide and subjected to colony count assay as described above. Assay buffer containing protease but not peptide and buffer containing untreated peptide were included as controls. Protease inhibition of the antimicrobial activity of CAMP peptide was expressed as a percentage of killing by protease treated peptide in relevance to the untreated peptide. The experiment was repeated three times in triplicate in each assay.

### Statistical analysis

Differences between treatment groups were analyzed using the one-way analysis of variance (ANOVA) followed by Duncan’s test for multiple comparisons (SPSS 19.0, IBM Corp., Armonk, NY). Statistical significance was indicated by *p* < 0.05.

## Results

### Peptide design

**Fig. 1:**
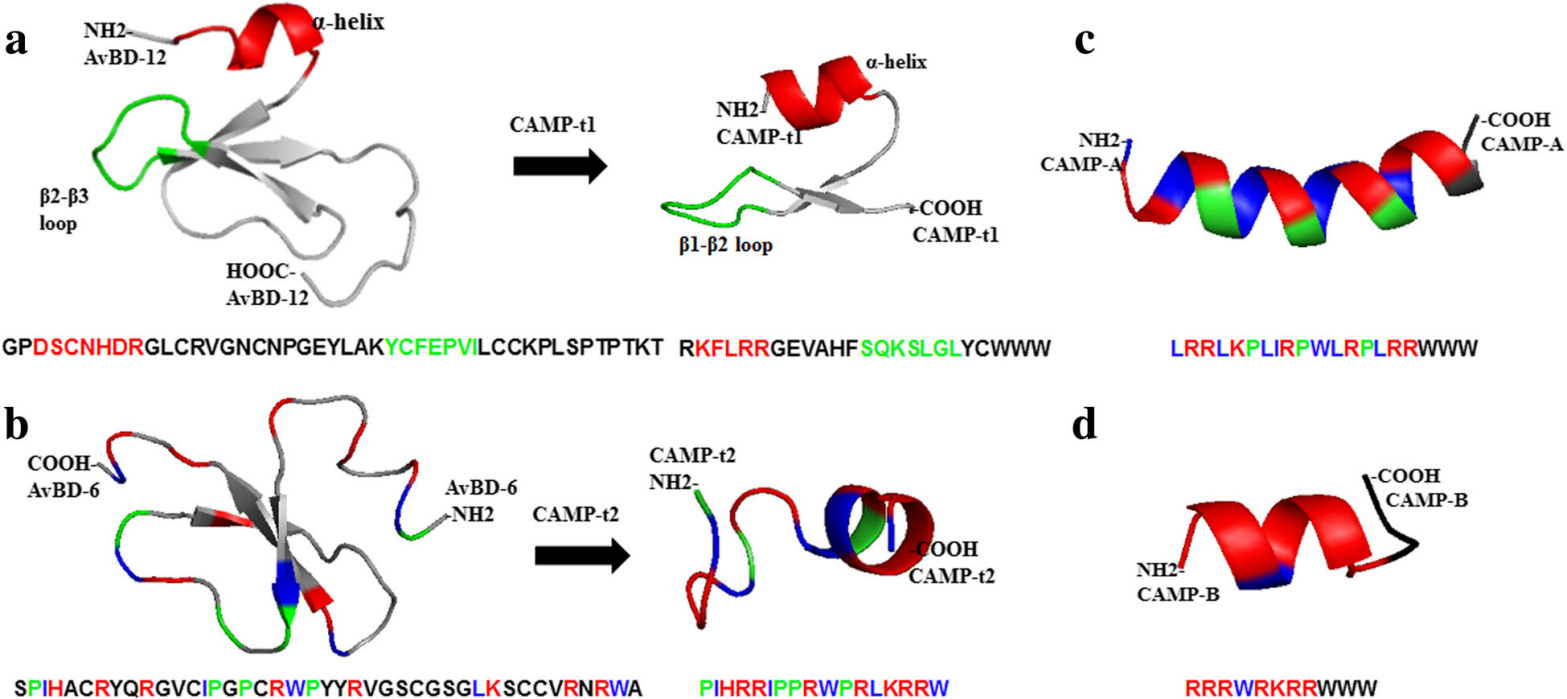
The predicted structures of newly designed templates CAMP-t1 and CAMP-t2. **a** The three-dimensional structure of CAMP-t1 derived from AvBD-12. Red: α-helix; Green: loop (β2-β3 loop in AvBD-12, β1-β2 loop in template CAMP-t1). **b** The three-dimensional structure of CAMP-t2 derived from AvBD-6. CAMP-t2 were further optimized to CAMP-A (**c**) and CAMP-B (**d**). CAMP-A and CAMP-B were coated with poly-Trp tails. Red: positively charged amino acid residues; Blue: hydrophobic amino acid residues; Green: prolines

Initially, two CAMP templates were designed to retain the antimicrobial and chemotactic properties of wild-type AvBD-6 and AvBD-12, respectively. The first CAMP template (CAMP-t1) possessed the structural domain (N-terminal α-helix and β2-β3 loop) of AvBD-12 as well as its analogues A2 and A3 which appeared to be essential to the broad chemotactic activity of AvBD-12 [13]. To increase net positive charge, the negatively charged amino acid residues Asp (D) and Glu (E) were substituted with positively charged amino acid residues Lys (K) or Arg (R). To enhance membrane permeabilization and salt resistance, a poly-Trp tail was incorporated to the C-terminus of the peptide, as it was previously shown that coating antimicrobial peptides with 3 Trp residues significantly increased salt resistance [18, 29, 35]. The resulting peptide CAMP-t1 consisted of 25 amino acid residues: RKFLRRRGEVAHFSQKSLGLYCWWW. The predicted three-dimensional structure of CAMP-t1 mimics that of AvBD-12 (Fig. 1a). The second CAMP template (CAMP-t2) consisted of the key amino acid residues of AvBD-6: PIHRRIPPRWPRLKRRW, responsible for the concentrated surface charge and hydrophobicity of AvBD-6 [13]. In silico analysis indicated that CAMP-t2 assumed a coil and α-helical structure (Fig. 1b). To optimize antimicrobial activity, CAMP-t2 was subjected to further modifications to create CAMP-A and CAMP-B (Fig. 1c and d) using the following criteria: 1) short in length (≤ 20 a.a. residues), 2) α-helical structure, 3) hydrophobic and hydrophilic residues on opposite sides of the helical surface to facilitate pore formation in bacterial membrane by multiple peptides [36], 4) lack of cutting sites for major proteases: aspartic protease, cysteine protease, metalloprotease, serine proteases, 5) N-terminal acetylation and C-terminal amidation to increase the metabolic stability of CAMPs [37], and 6) addition of C-terminal poly-Trp tail to enhance membrane permeabilization and salt resistance [29, 35].

### Structural features

**Fig. 2:**
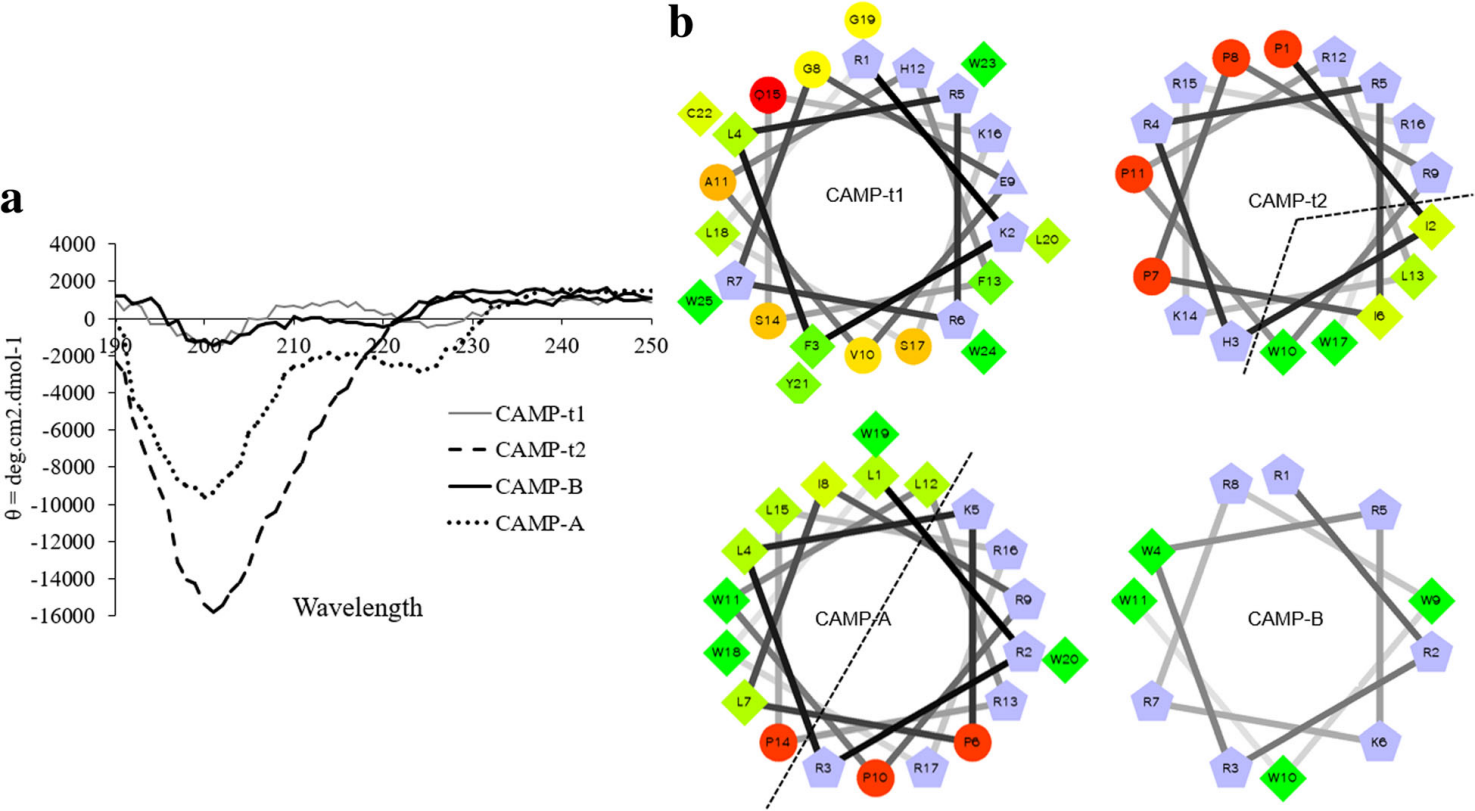
The far-UV CD spectra and helical wheels projections of CAMPs. **a** The far-UV CD spectra of CAMPs in H2O recorded at room temperature. Spectra were baseline corrected and expressed as molar ellipticity θ (deg·cm^2^·mol^− 1^). Gray solid line: CAMP-t1; dash line: CAMP-t2; dotted line: CAMP-A; dark solid line: CAMP-B. **b** Helical wheels projections of CAMPs. Relevant features of amino acid residues were coded by various shapes and colors. Hydrophilic residues: circles, hydrophobic residues: diamonds, positively charged residues: pentagons. Hydrophobic residues: green to yellow, as the hydrophobicity decreased to zero, color changed gradually from dark green to yellow. Hydrophilic residues: red, the red tone decreased proportionally to the decrease in hydrophilicity. Charged residues: light blue

The amino acid sequences and relevant biochemical characteristics of all CAMPs were presented in Table 1. The structures of CAMPs was analyzed by a far-UV spectrometer. In general, the CD spectrum of α-helical structures presents two negative bands at 208 and 222 nm along with a positive band at 192 nm whereas random coil is characterized by a single band below negative 200 nm [38]. As shown in Fig. 2a, CAMP-t1 showed two weak bands at 202 nm and 225 nm, indicating partial α-helical structure of the peptide. CAMP-t2 displayed a strong single band around 200 nm, confirming the random coil structure as predicted in silico (Fig. 1b). CAMP-A had two negative bands at 200 nm and 225 nm along with a positive band at 190 nm which verified the predicted α-helical structure. CAMP-B showed two weak bands at 202 nm and 225 nm which was consistent with the predicted partial α-helix (Fig. 1d). The CAMPs were subjected to helical wheels projection analysis (Fig. 2b). CAMP-t1 showed random distribution of amino acids and CAMP-t2 showed a large distribution angle of hydrophilic residues (proline and positively charged amino acids) and a small distribution angle of hydrophobic residues. CAMP-A displayed an amphipathic structure with hydrophobic and hydrophilic residues on the opposite site of the helix, which supported the designing feature (Figs. 1c and 2b). CAMP-B exhibited a small helical wheel with hydrophobic residue W4 in the middle of positively charged residues (Fig. 1d) and the W9W10W11-tail folding along with the helix.

**Table 1:** The characteristics of newly designed CAMPs

Peptide^a^	Amino acid sequence	Length (aa)	Molecular Weight	Charge	Hydrophobicity
CAMP-t1	RKFLRRRGEVAHFSQKSLGLYCWWW	25	3251.84	+ 4	44%
CAMP-t2	PIHRRIPPRWPRLKRRW	17	2361.90	+ 7	29%
CAMP-A	LRRLKPLIRPWLRPLRRWWW	20	2839.55	+ 7	50%
CAMP-B	RRRWRKRRWWW	11	1869.24	+ 7	36%

^a^CAMP-t1, CAMP-t2, and CAMP-B are coated with a Trp-tail. N-terminal acetylation and C-terminal amidation were introduced to all peptides

### Antimicrobial activity

The minimum inhibitory concentrations of the newly designed CAMPs against *P. aeruginosa* and *S. aureus* ATCC reference strains and multidrug-resistant *P. aeruginosa* and methicillin-resistant *S. pseudinetermediu*s (MASP) strains were compared to that of AvBD-6, a natural host CAMP with potent antimicrobial activity under low salt conditions (Table 2). CAMP-t1 showed significantly improved anti-*Pseudomonas* activity with MIC values 4-fold (for ATCC reference strain) and 2-fold (clinical isolates) lower than that of AvBD-6. CAMP-t1 showed improved antimicrobial activity against *S. aureus* ATCC reference strain (MIC was 4-fold lower than that of AvBD-6), but not MRSP clinical isolates as evidenced by the high MIC values similar to that of AvBD-6. CAMP-t2, the shorter template, demonstrated similar antimicrobial activity against *Pseudomonas* strains and significantly enhanced anti-*Staphylococcus* activity, compared to CAP-t1. The MICs of CAMP-t2 against *S. aureus* and MRSP strains were up to 4-fold lower than that of CAMP-t1 and AvBD-6. CAMP-A, a derivative of CAMP-t2, established further improved antimicrobial activity against *P. aeruginosa* and *S. aureus* reference strains, multidrug-resistant *P. aeruginosa* strains and MRSP clinical isolates. The MICs of CAMP-A against *P. aeruginosa* and MRSP isolates were 4- to 32- fold lower than that of AvBD-6. CAMP-B, the second derivative of CAMP-t2, also showed improved antimicrobial activity against *P. aeruginosa* and similar potency against *Staphylococcus* spp. However, CAMP-B was less effective than CAMP-A in killing both *Pseudomonas* spp. and methicillin-resistant *S. pseudintermedius* (*p* < 0.05, Table 2).

**Table 2:** The minimum inhibitory concentration (MIC) of CAMPs

CAMPs	CAMP-t1	CAMP-t2	CAMP-A	CAMP-B	AvBD-6
Bacteria (strain identification)	MIC (μg/ml)	MIC (μg/ml)	MIC (μg/ml)	MIC (μg/ml)	MIC (μg/ml)
*P. aeruginosa* (ATCC 27853)	64	64	16	32	> 256
*P. aeruginosa* (1704173) ^a^	128	64	16	32	> 256
*P. aeruginosa* (1703357) ^a^	128	128	16	64	> 256
*P. aeruginosa* (1703511) ^a^	128	128	16	64	> 256
*P. aeruginosa* (1703000) ^a^	128	64	8	32	> 256
*P. aeruginosa* (1703002) ^a^	128	256	16	64	> 256
*P. aeruginosa* (1703451) ^a^	128	64	16	64	> 256
*P. aeruginosa* (1703949) ^a^	128	128	16	64	> 256
*P. aeruginosa* (1703290) ^a^	128	128	16	64	> 256
*P. aeruginosa* (1703983) ^a^	128	256	16	64	> 256
*P. aeruginosa* (1704175) ^a^	128	64	16	32	> 256
MIC_Average G-_	122.18 ± 19.29^a^	122.18 ± 72.71^a^	15.27 ± 2.41^c^	52.36 ± 16.14^b^	> 256
*S. aureus* (ATCC 29213)	64	64	32	32	256
*S. pseudintermedius*(13164006)^b^	256	32	16	32	> 256
*S. pseudintermedius*(13203008)^b^	256	32	16	64	256
*S. pseudintermedius*(13178007)^b^	256	64	16	64	256
*S. pseudintermedius*(13267017)^b^	128	32	16	32	> 256
*S. pseudintermedius*(13252001)^b^	64	32	16	32	256
*S. pseudintermedius*(13269013)^b^	256	32	32	32	> 256
*S. pseudintermedius*(13193006)^b^	256	128	32	32	> 256
*S. pseudintermedius*(13228005)^b^	256	32	16	64	> 256
*S. pseudintermedius*(13207007)^b^	256	32	16	64	> 256
*S. pseudintermedius*(13250111)^b^	256	32	32	64	256
MIC_Average G+_	209.45 ± 81.41^a^	46.55 ± 29.89^b^	21.82 ± 8.07^c^	46.55 ± 16.71^b^	≥256

^a^Multidrug resistant clinical isolates of *P. aeruginosa* were resistant to chloramphenicol, tetracycline, sulfamethoxazole, and β-lactam antibiotics: amoxicillin, ampicillin and cefazolin.

^b^Methicillin-resistant *S. pseudintermedius* (MRSP)

Superscripts a, b, and c mean significant difference (*p* < 0.05) between MICs of four CAMPs against either Gram-negative or Gram-positive bacteria

### Membrane permeabilizing activity

**Fig. 3:**
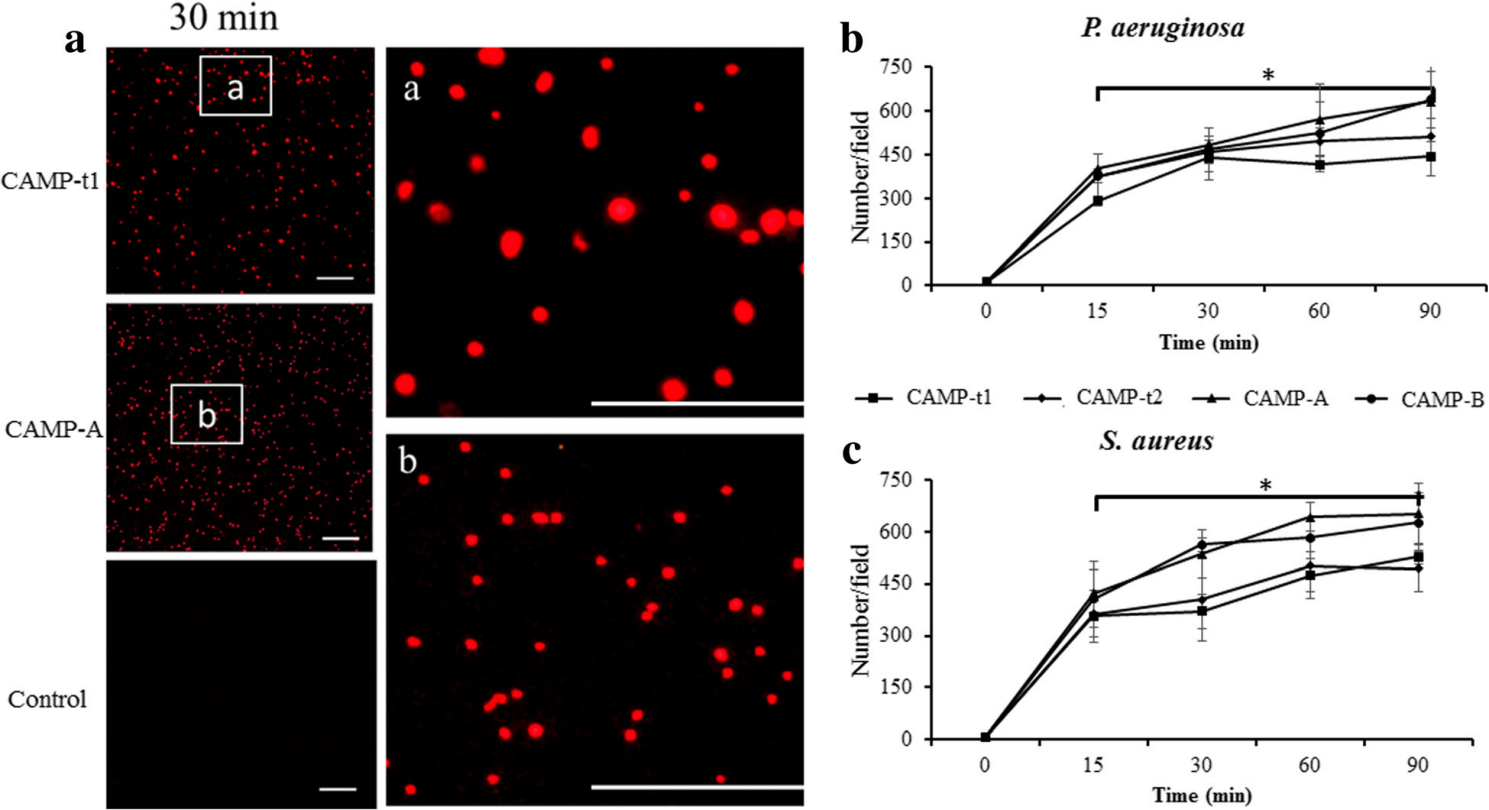
Membrane permeabilizing activity of CAMPs. **a** Representative fluorescence microscopy images of CAMP-treated and control bacteria stained with membrane-impermeable DNA dye propidium iodide (PI). Right panels are enlarged focal areas (**a** and **b**) from the left panels. **b** The number per field of positively stained *P. aeruginosa* at various times post-CAMP-treatment. **c** The number per field of *S. aureus* at various times post-CAMP-treatment. Data are expressed as the means ± SD of three independent experiments. An asterisk indicates a significant difference between different time points (*p < 0.05). Bar: 100 μm

A propidium iodide (PI) uptake assay was carried out to determine the membrane permeabilizing activity of newly designed CAMPs (Fig. 3). As shown in Fig. 3a, CAMP-treated *P. aeruginosa* were stained red, indicating that bacterial membranes were damaged by CAMPs. In contrast, untreated bacteria did not show red fluorescence. When the permeabilizing ability was assessed based on the numbers of red cells per field, a time-dependent increase was observed from 15 to 90 min (Fig. 3b and c). However, the number of red bacteria did not increase significantly after 30 min, indicating a fast-action mode of CAMPs. Similar results were obtained for all CAMPs and both bacterial pathogens (Fig. 3).

### Salt resistance

**Fig. 4:**
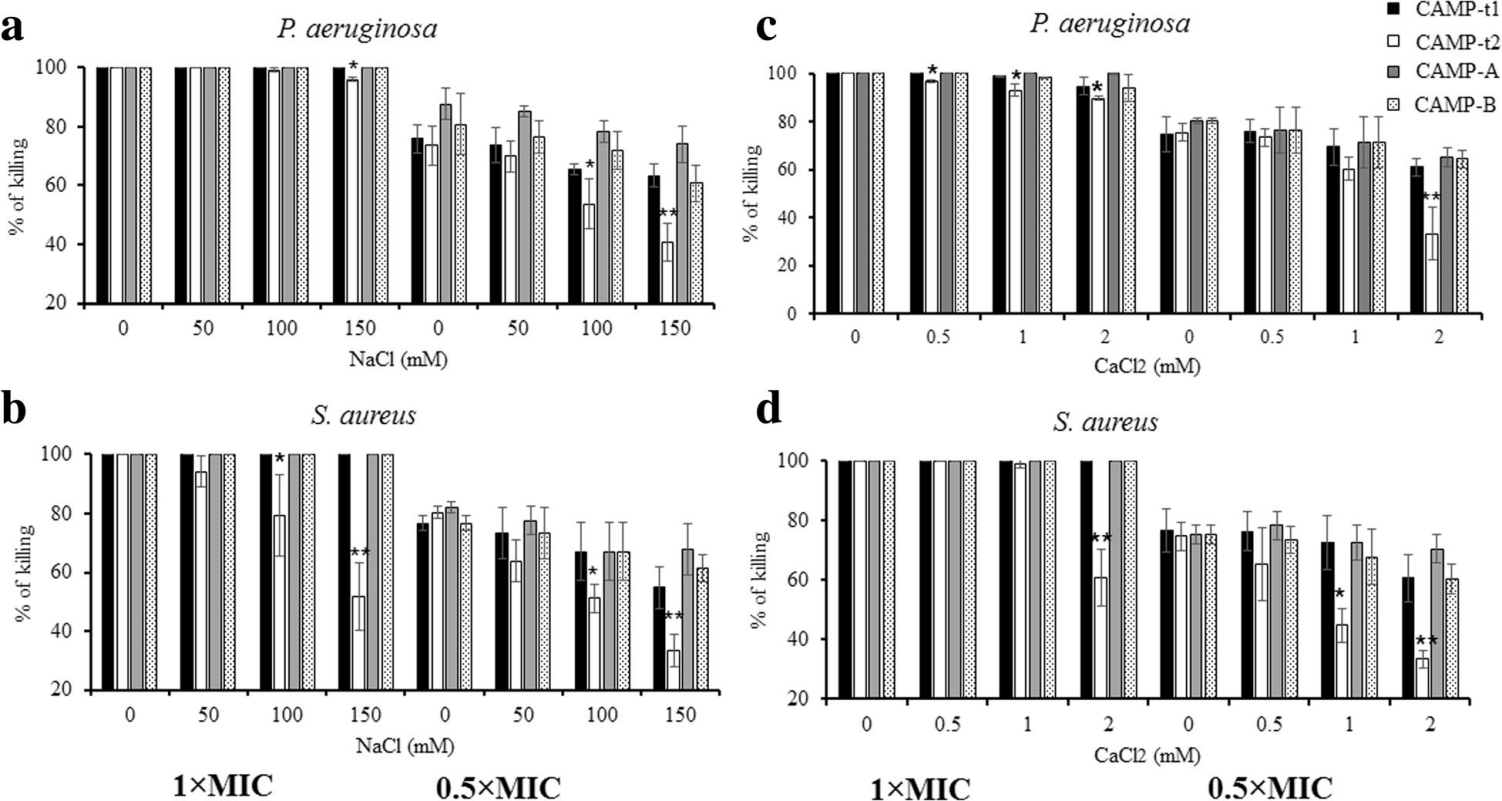
Effect of salts on the antibacterial activity of CAMPs against *P. aeruginosa* and *S. aureus*. The effect of salts on the antibacterial activity was determined using two peptide concentrations: 1 × MIC and 0.5 × MIC. **a** Percent of killing against *P. aeruginosa* at 0, 50, 100, and 150 mM NaCl; (**b**) Percent of killing against *S. aureus* at 0, 50, 100, and 150 mM NaCl; (**c**) Percent of killing against *S. aureus* at 0, 0.5, 1, and 2 mM CaCl_2_. **d** Percent of killing against *S. aureus* at 0, 0.5, 1, and 2 mM CaCl_2_. Data represent the means ± SD of three independent experiments. An asterisk indicates the statistically significant difference between antimicrobial activity in the presence and absence of salts (*p < 0.05 and **p < 0.01)

The impact of cationic salts on the bactericidal activity of CAMPs was assessed using an assay system containing various concentrations of NaCl (0 to 150 mM) or CaCl_2_ (0 to 2 mM) at two peptide concentrations (1 × MIC and 0.5 × MIC). As shown in Fig. 4, increasing NaCl and CaCl_2_ concentrations had no impact on the bactericidal activity of CAMP-t1 which possesses a C-terminal poly-Trp tail. In contrast, the bactericidal activity of CAMP-B without the Trp tail was negatively affected by increased salt concentrations. At the physiological conditions of NaCl (100 to 150 mM) and CaCl_2_ (1 to 2 mM), CAMP-t2 retained approximately 40% of its killing activity against *P. aeruginosa* and *S. aureus*, compared to the results obtained at salt-free condition (Fig. 4). The salt-resistance pattern of CAMP-t2 was similar at two different peptide concentrations. Similar to CAMP-t1, CAMP-A and CAMP-B with a Trp tail exhibited strong tolerance to NaCl and CaCl_2_ (Fig. 4).

### Hemolytic activity and cytotoxicity

**Fig. 5:**
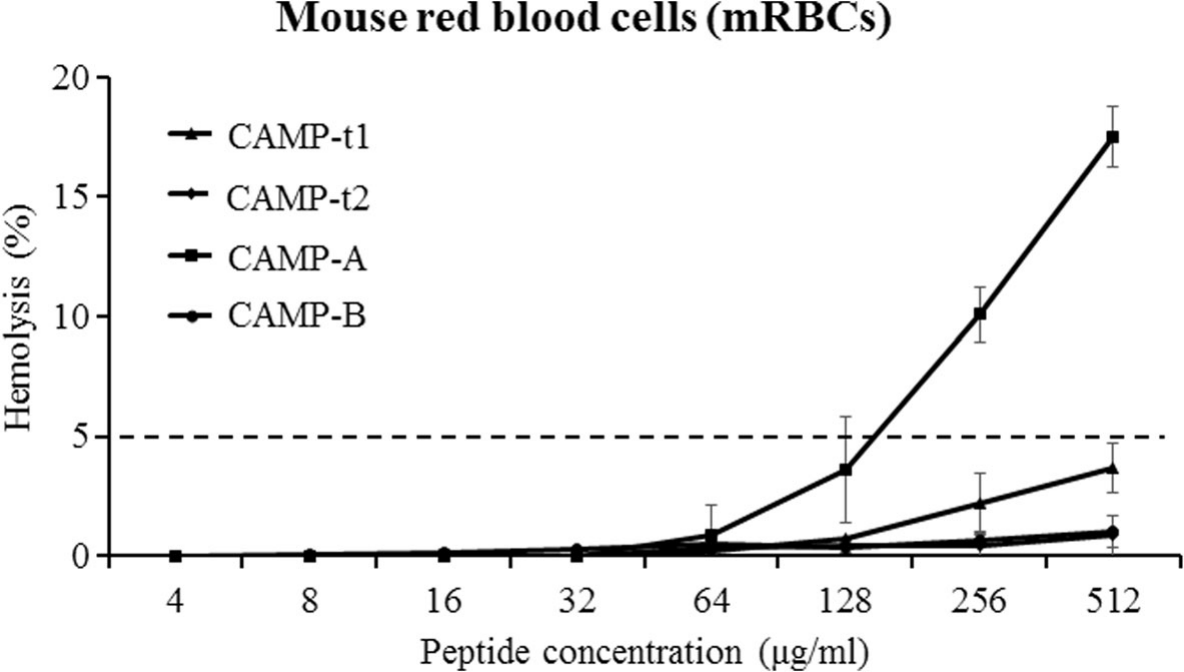
Hemolytic activity of CAMPs. CAMP-induced hemolysis (%) of mouse red blood cells at various peptide concentrations is defined as a percentage of complete hemolysis caused by 0.2% Triton X-100. Data are expressed as the means ± SD of three independent experiments

The hemolytic activity of CAMPs to mouse RBCs was analyzed (Fig. 5). At high concentrations, CAMP-A lysed approximately 3.6% of mRBCs at 128 μg/ml, 10.1% of mRBCs at 256 μg/ml, and 17.5% of mRBCs at 512 μg/ml. CAMP-t1, CAMP-t2, and CAMP-B did not cause more than 5% of mRBCs at the concentration of 512 μg/ml. The minimum hemolytic concentration (MHC), geometric means of the MIC (MIC_GM_), and therapeutic index (T.I.) were determined for each CAMP (Table 3). The average T.I. of CAMP-A and CAMP-B against *P. aeruginosa* were 8.72 ± 2.41 and > 10.90 ± 4.0, respectively. The T.I. of CAMP-A and CAMP-B against *S. aureus* were 6.54 ± 2.01 and > 12.36 ± 4.18, respectively.

**Table 3:** Therapeutic index of new cationic antimicrobial peptides (CAMPs)

Peptides	CAMP-t1	CAMP-t2	CAMP-A	CAMP-B
MHC	> 512	> 512	128	> 512
MIC_Average G-_	122.18 ± 19.29	122.18 ± 72.71	15.27 ± 2.41	52.36 ± 16.14
MIC_Average G+_	209.45 ± 81.41	46.55 ± 29.89	21.82 ± 8.07	46.55 ± 16.71
T.I. of G-	> 4.36 ± 1.21	> 5.45 ± 2.54	8.72 ± 2.41	> 10.90 ± 4.0
T.I. of G+	> 3.27 ± 2.41	> 13.45 ± 4.48	6.54 ± 2.01	> 12.36 ± 4.18

Therapeutic index, (*T.I*.) is defined as the ratio of, *MHC* to, *MIC*_*GM*_. *MHC* (μg/ml) is the minimum hemolytic concentration that caused 5% hemolysis of mouse red blood cells, (*mRBCs*). The MIC_GM_ (μg/ml) means the geometric mean, (*GM*) of the, *MIC* values of the peptides against bacteria. *MIC*_*Average G*-_ is the, *MIC*_*GM*_ for Gram-negative bacteria, *MIC*_*Average G+*_ is the MIC_GM_ for Gram-positive bacteria

**Fig. 6:**
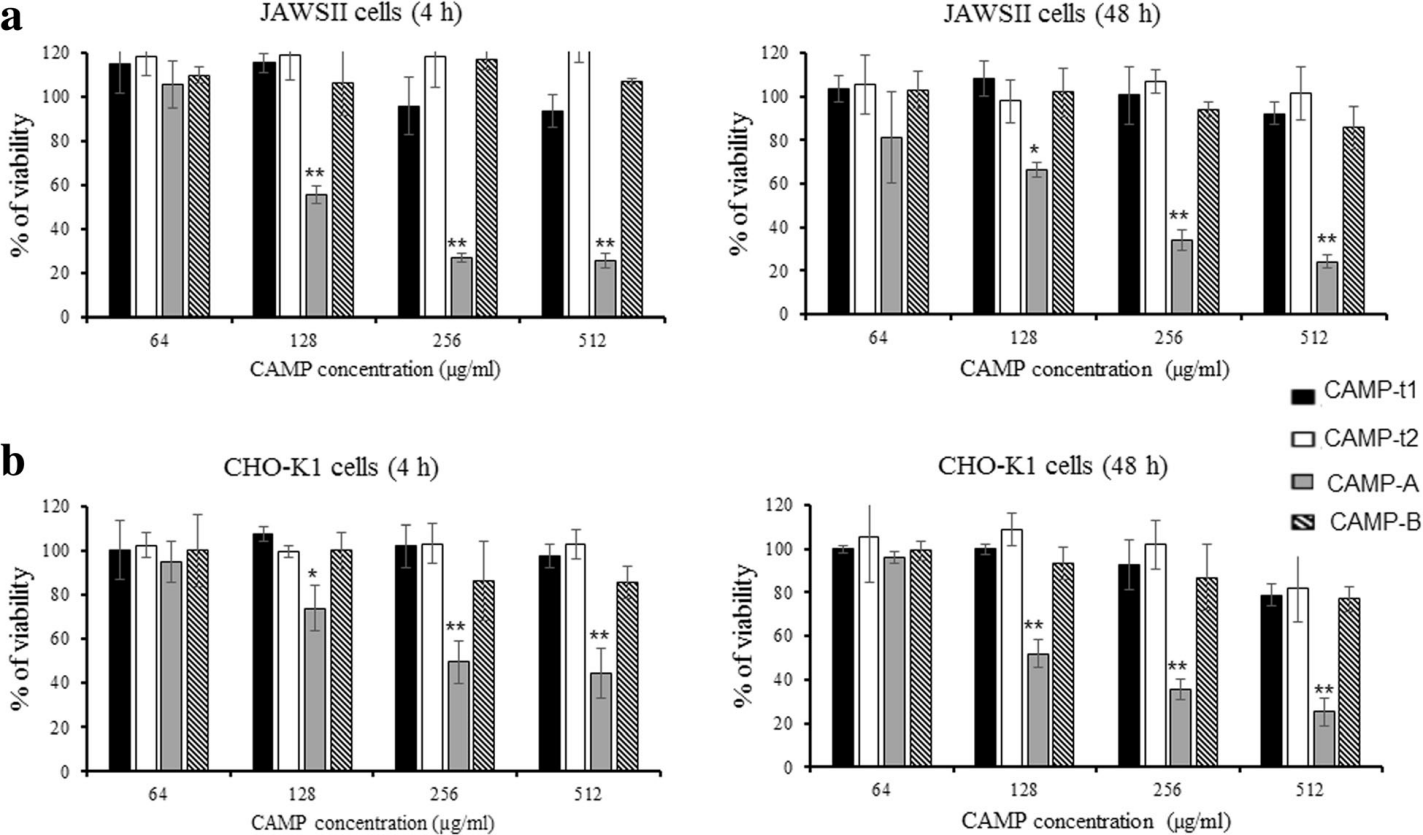
Cytotoxicity of CAMPs to JAWSII and CHO-K1 cells. Effect of CAMPs on the viability of mouse immature dendritic JAWSII cells (**a**) and hamster ovary CHO-K1 cells (**b**) at 4 and 48 h of incubation with peptide at the concentration of 64 to 512 μg/ml. Results are percentages of viable cells relative to the untreated control cells. The data are expressed as the mean ± SD of three independent experiments. An asterisk indicates a statistically significant difference in the viability of CAMP-treated cells and untreated cells (*p < 0.05 and **p < 0.01)

Cell cytotoxicity of the newly designed CAMPs was also determined by MTT cell viability assay (Fig. 6). Exposure of murine immature dendritic cell line JAWSII (Fig. 6a) and CHO-K1 (Fig. 6b) cells to CAMP-t1, CAMP-t2, and CAMP-B at concentrations of 64, 128, 256, and 512 μg/ml for 4 to 48 h did not significantly affect cell viability. However, the viability of both JAWSII and CHO-K1 cells was significantly decreased by treatment with CAMP-A at a concentration equal to or greater than 128 μg/ml. After treatment with CAMP-A, no significant difference in the percentage of cell viability was observed between 4 h treatment and 48 h treatment.

### Chemotactic activity

**Fig. 7:**
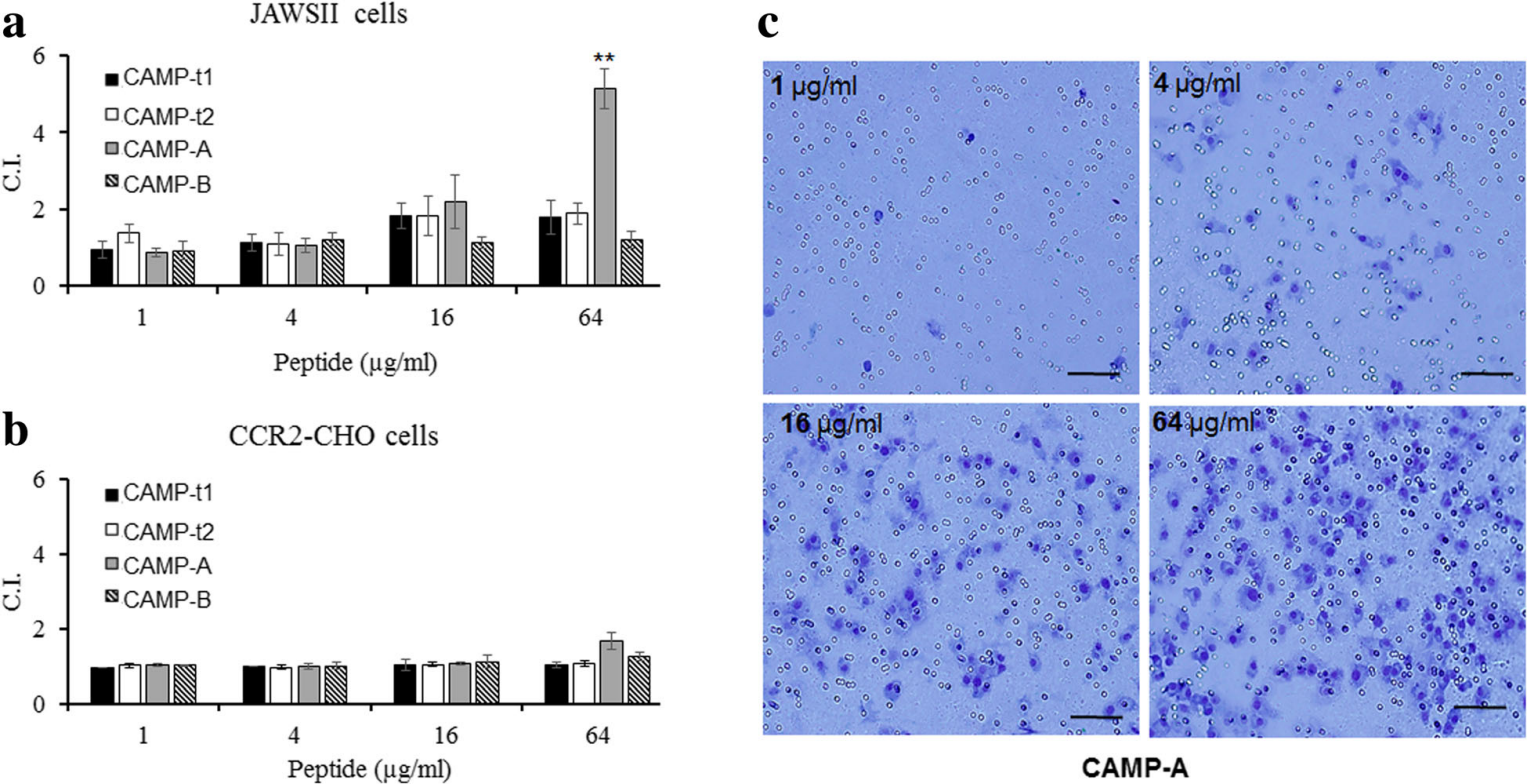
Chemotactic activity of CAMPs. CAMP-induced migration of mouse immature dendritic JAWSII cells (**a**) and CHO-K1 cells expressing avian CCR2 (**b**) was measured at the following peptide concentrations: 1, 4, 16, and 64 μg/ml. **c** Migrated JAWSII cells on the membrane induced by CAMP-A at peptide concentrations of 1, 4, 16, and 64 μg/ml. White pores are membrane pores (diameter: 8 μm) and blue ones are stained JAWSII cells. Bar: 50 μm. Chemotactic index (C.I.) was expressed as the number of migrated cells induced by CAMP / the number of migrated cells in response to chemotactic buffer. Data represent the means of five independent experiments ± SD. An asterisk indicates significant difference (*p < 0.05 and **p < 0.01)

The chemotactic activity of the CAMPs for JAWSII and CCR2-transfected CHO-K1 cells were determined (Fig. 7). The results indicated that CAMP-t1 with the N-terminal helix-loop structure of AvBD-12 did not show expected chemotactic activity to either cell line (Fig. 7a and b). Interestingly, CAMP-A with the highest antimicrobial activity showed mild chemotactic activity at a concentration of 64 μg/ml (C.I. = 5.13; 77.5% of wild-type AvBD-12, C.I. = 6.62) for JAWSII cells. As shown in Fig. 7c, more JAWSII cell migration was induced by CAMP-A with increasing peptide concentrations, ranging from 1 to 64 μg/ml. No significant chemotactic activity for CCR-2 transfected CHO-K1 cells was detected (Fig. 7b). CAMP-t2 and CAMP-B did not show any chemotactic activity for either JAWSII or CCR2-CHO-K1 cells.

### Protease resistance

**Fig. 8:**
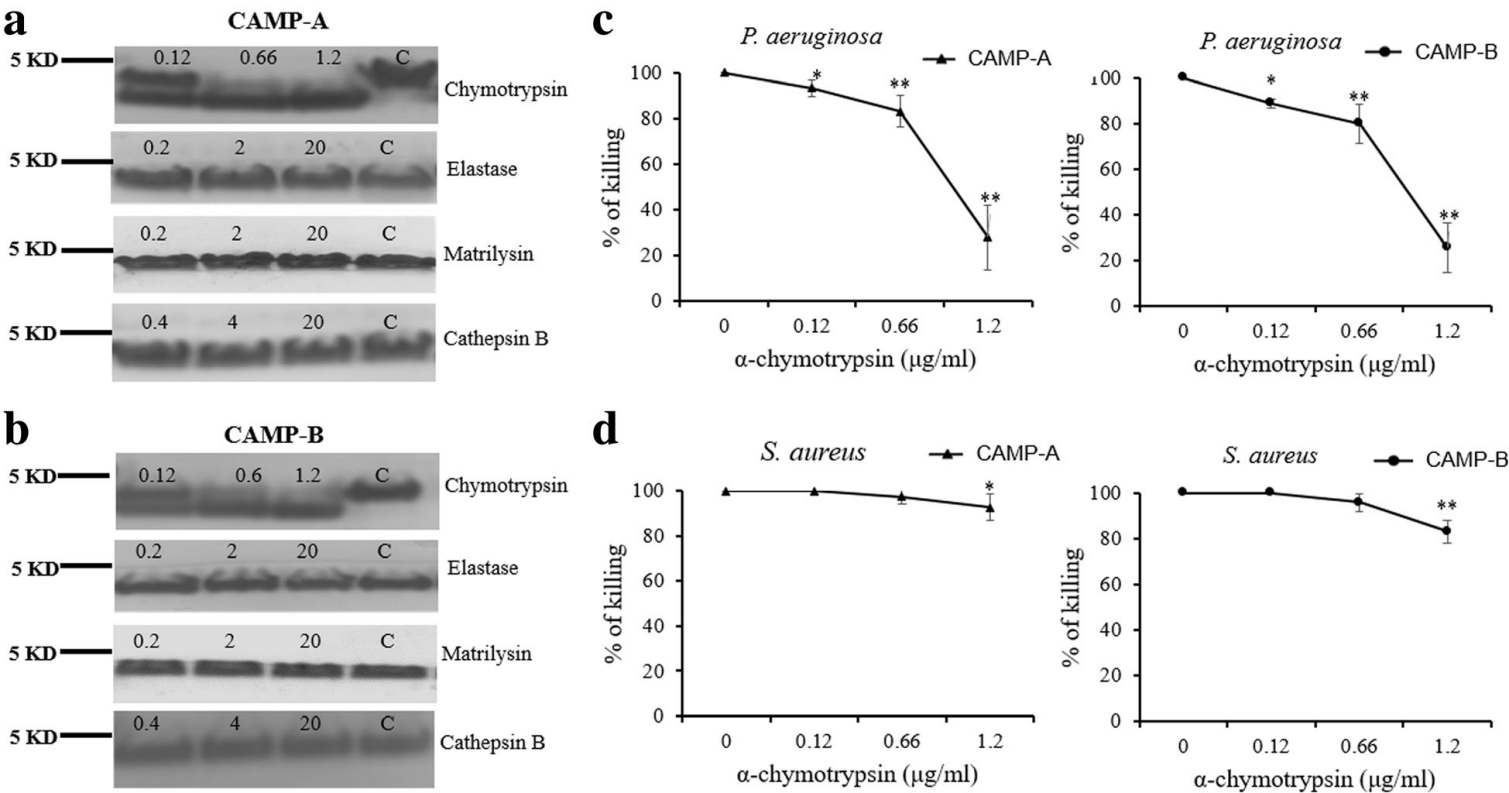
Effects of protease treatment on the antimicrobial activity of CAMP-A and CAMP-B against *P. aeruginosa* and *S. aureus*. Peptides were digested with α-chymotrypsin, elastase, matrilysin, or cathepsin B for 1 h at 37 °C and subjected to SDS-PAGE (16.5% polyacrylamide gel) analysis. **a** CAMP-(**a**). **b** CAMP-(**b**). **c** Antimicrobial activity of CAMP-A post-digestion by α-chymotrypsin at the concentration of 0.12, 0.66, and 1.2 μg/ml. **d** Antimicrobial activity of CAMP-B post-digestion by α-chymotrypsin at the concentration of 0.12, 0.66, and 1.2 μg/ml. Results are expressed as percent killing by digested peptides over untreated peptides. Data are expressed as the means ± SD of three independent experiments. An asterisk indicates a statistically significant difference between antimicrobial activity with and without protease (*p < 0.05 and **p < 0.01)

The resistance of CAMP-A and CAMP-B, two peptides exhibiting strong antimicrobial activity and salt-resistance, to various proteases was evaluated by subjecting protease-treated peptides to SDS-PAGE (Fig. 8). The results indicated that CAMP-A and CAMP-B were partially digested by α-chymotrypsin at 0.12 to 1.2 μg/ml, as indicated by the presence of peptide bands with lower molecular weight than that of the untreated peptides (Fig. 8a and b). The antimicrobial activity of CAMP-A and CAMP-B against *P. aeruginosa* decreased significantly post-digestion (Fig. 8c). The digested peptides retained approximately 90% (at 0.12 μg/ml), 80% (at 0.66 μg/ml), and 30% (at 1.2 μg/ml) of the killing activity of the untreated CAMP-A or CAMP-B. In contrast, the anti-*Staphylococcus* activity was mildly affected only when the CAMPs were treated with the highest concentration (1.2 μg/ml) of α-chymotrypsin (Fig. 8d). CAMP-A and CAMP-B were not cleaved by metalloproteinases matrilysin, elastase, and cathepsin B at concentrations up to 20 μg/ml. Treatment with these proteases did not affect the antimicrobial activity of CAMP-A and CAMP-B (data not shown).

## Discussion

Host cationic antimicrobial peptides, such as defensins, have been a subject of research interest because of their broad-spectrum antimicrobial activity and low potential for resistance development. For instance, human α-defensin HD5 and HDP4 showed strong killing activity against *S. aureus* ATCC 25923 and ATCC 29213 with the lethal doses [39]. Our previous structure-function analysis of AvBDs and their analogues indicates that the highly concentrated surface positive charge plays a predominant role in the antimicrobial potency of the peptides whereas the CCR2-binding domain (N-terminal α-helix along with an adjacent loop) is responsible for the broad-spectrum chemotactic activity for both avian and mammalian dendritic cells [13]. For example, linear AvBD analogues with a high net positive charge (+ 9), modest hydrophobicity (40%), and a predicted CCR2 binding domain exhibit strong antimicrobial and mild chemotactic activities. However, the linear peptides designed in our previous study are still lengthy (45 amino acid residues) and susceptible to physiological concentrations of NaCl. Although the sensitivity of AvBD analogues to proteases was not evaluated in our previous studies, investigations conducted by others suggest that linear peptides are susceptible to bacterial metalloprotease, cysteine protease and human neutrophil elastase [38, 39]. To develop antimicrobial peptides suitable for therapeutic or preventive use, we utilized an integrated approach to modify AvBD analogues to achieve the following goals: structurally simple (linear, short and all natural amino acids), resistant to proteases and cationic salts, non-cytotoxic, strong and broad-spectrum antimicrobial activity, and potentially chemotactic for immune cells.

We first designed two CAMP templates, CAMP-t1 and CAMP-t2, by extrapolating the CCR2 binding domain of AvBD-12 (CAMP-t1) and key amino acid residues contributing to the concentrated surface positive charge and hydrophobicity of AvBD-6 (CAMP-t2), respectively. For CAMP-A, the negatively charged amino acid residues (D and E) in AvBD-12 were replaced by positively charged residues (K and R). Because the net positive charge of CAMP-t1 was still relatively low (+ 4), a poly-Trp tail was incorporated into its C-terminus to boost the antimicrobial activity. Trp is known for its tendency to insert into membrane lipid bilayer and Trp-rich peptides exhibit enhanced antimicrobial activity and salt resistance [18]. N-terminal acetylation and C-terminal amidation (mimicking native proteins) were also incorporated to increase the metabolic stability of peptides as well as their resistance to enzymatic degradation [40]. To evaluate the antimicrobial properties of these peptides, we determined their MICs against *P. aeruginosa* and *Staphylococcus* according to the guidelines of CLSI [27, 28]. Both CAMP-t1 and CAMP-t2 demonstrated improved antimicrobial activity, compared to AvBD-6 and AvBD-12 as well as previously designed AvBD analogues [13, 14]. Although CAMP-t1 retained the N-terminal α-helix and an adjacent loop structure of AvBD-12, it lost the desired chemotactic property [13], suggesting that either the amino acid composition was not optimal or additional structural components were required for CCR2 binding. We then focused on CAMP-t2, a shorter template with a coil-helix structure. This peptide showed stronger antimicrobial activity against methicillin-resistant S. *pseudintermedius* than CAMP-t1 and AvBD-6, but was sensitive to high concentrations of cationic salts. The rest of the study was concentrated primarily on improving the antimicrobial property and salt resistance of CAMP-t2.

Studies have indicated that amphipathicity is a key characteristic required for membrane permeabilization in which hydrophobic residues interact with membrane lipid components while hydrophilic regions either bind with the phospholipid head groups or form the lumen of a membrane pore [5, 14]. Alpha-helical peptides with hydrophobic and hydrophilic residues on opposite sides of the peptide molecule have antimicrobial property [8]. The shorter template, CAMP-t2 with coil-helix structure (only 35% of residues form α-helix), was further modified to form an α-helix structure which confers structural stability [41]. To maximize the antimicrobial activity and minimize the damaging effect on host cell membrane, Trp and Pro residues were incorporated and the amino acid residues were strategically arranged to avoid protease cutting sites predicted using online PROSPER and SignalP 4.1 servers. The resulting peptides, CAMP-A and CAMP-B, demonstrated strong antimicrobial activity against ATCC bacterial reference strains as well as multi-drug resistant *P. aeruginosa* and methicillin-resistant *S. pseudintermedius* clinical isolates. With a poly-Trp tail, α-helical structure and increased surface positive charge, CAMP-A and CAMP-B were fully functional at physiological concentrations of NaCl and CaCl_2_. These peptides were also resistant to metalloproteinases, matrilysin and elastase, and cathepsin B at concentrations higher than that in bacterial protein secretion [42] or in mammalian host cells [34]. Although they were still cleaved by α-chymotrypsin, the antimicrobial activity was minimally affected at the concentration of 0.12 μg/ml, about 3 to 40 times higher than the concentration tested in human samples using different methods, 4 ng/ml [43] and 37.5 ng/ml [44]. At a high concentration (1.2 μg/ml), α-chymotrypsin treatment reduced the killing activity against *P. aeruginosa* (*p* < 0.05) but not *S. aureus* (*p* > 0.05). The discrepancy could be associated with structural difference between Gram-negative and Gram-positive bacterial membranes which warrants further investigation into the mechanism of antimicrobial action of these peptides.

Our previous studies have shown that AvBDs could disrupt bacterial membrane resulting in cell deformation, increased membrane permeabilization, and membrane damage [13, 14]. In the present study, data from propidium iodide (PI) staining assay suggested that the primary mode of action of the newly designed CAMPs was membrane attacking, which is considered a mechanism less likely to trigger bacterial resistance. The CAMPs did not show any detectable cytotoxicity and hemolytic activity at the doses required for effective bacterial killing. CAMP-t1, CAMP-t2, and CAMP-B had minimal cytotoxic and hemolytic activities at a relatively high peptide concentration (512 μM/ml). CAMP-A, the most potent antimicrobial peptide, exhibited hemolytic and cytotoxic activities at concentrations equal to or greater than 128 μg/ml which, however, was 6-fold higher than the MIC against *P. aeruginosa* and 4-fold higher than the MIC against *Staphylococcus* spp*.* The cytotoxic property of CAMP-A was not surprising because the peptide had a relatively high hydrophobicity (50%), which is known hydrophobicity is associated with their cytotoxic effect [45]. The undesired hemolytic activity was still mild compared to other antimicrobial peptides including magainin isolated from the skin of African frog *Xenopus laevis* and melittin from bee venom [46].

It has been suggested that the three conserved disulfide bridges were required for the chemotactic function of β-defensins [14, 47–49]. Data from our previous study indicated that a predicted CCR2 binding domain (N-terminal α-helix and an adjacent β2-β3 loop) in AvBD-12A3 (a linear peptide) without disulfide bridges was chemotactic to JAWSII cells [13]. In the present study, CAMP-t1 with a similar helix-loop domain failed to show chemotactic activity. Interestingly, CAMP-A with high positive charge and modest hydrophobicity induced chemotactic migration of JAWSII cells which occurred possibly via the formyl-peptide receptors like mechanism such reported for human cathelicidin LL-37 [50] and cathelicidin-like pleurocidins [51].

## Conclusion

CAMP-t1 and CAMP-t2 were designed as templates based on key structural and functional components of AvBD-12 and AvBD-6. CAMP-t1 with a predicted CCR binding domain of AvBD-12 demonstrated improved antimicrobial activity but lost the original chemotactic function. CAMP-t2 with key amino acid residues of AvBD-6 showed strong antimicrobial activity, but sensitivity to high concentrations of cationic salts. CAMP-t2 was further modified using an integrated design approach. CAMP-A and CAMP-B possess the following advantageous characteristics: structural simplicity (short and linear), resistance to salts and proteases, potent antimicrobial activity against multidrug-resistant *P. aeruginosa* and methicillin-resistant *Staphylococcus*, rapid membrane attacking mode, and moderate therapeutic index. Our data suggest that CAMP-A and CAMP-B are excellent candidates for development as antimicrobial therapeutic agents.

## Abbreviations

ANOVA: One-way analysis of variance
AvBD: Avian beta-defensin
BSA: Bovine serum albumin
CFU: Colony-forming unit
CI: Chemotaxis index
DCs: Dendritic cells
ESI-MS: Electrospray ionization mass spectrometry
FBS: Fetal bovine serum
fMLF: N-Formyl-methionyl-leucyl-phenylalanine
Fmoc: 9-fluorenyl-methoxycarbonyl
GM-CSF: Granulocyte macrophage colony-stimulating factor
hBD6: Human β-defensin 6
LB: Luria-Bertani
LPS: Lipopolysaccharides
LTA: Lipoteichoic acid
MIC: Minimum inhibitory concentration
PBS: Phosphate buffered saline
PI: Propidium iodide
RP-HPLC: Reversed-phase high-performance liquid chromatography
SD: Standard deviation
